# Primary CNS Lymphoma

**DOI:** 10.1007/s11899-014-0217-2

**Published:** 2014-06-27

**Authors:** Elizabeth H. Phillips, Christopher P. Fox, Kate Cwynarski

**Affiliations:** 1 Department of Haematology, Royal Free Hospital, Pond St, London, UK; 2 Department of Clinical Haematology, Nottingham University Hospitals NHS Trust, Nottingham, UK

**Keywords:** CNS lymphoma, Pathobiology, Imaging, Clinical management, Lymphoid malignancy

## Abstract

Primary diffuse large B-cell lymphoma (DLBCL) of the central nervous system is an aggressive malignancy that exhibits unique biological features and characteristic clinical behaviour, with overall long-term survival rates of around 20–40 %. Clinical outcome has improved following the advent of chemoradiation protocols incorporating high-dose methotrexate in the mid-1980s, but disease relapse and adverse neurocognitive sequelae remain major clinical challenges. To address this, investigators have focused on improving drug therapy with novel cytotoxic combinations, monoclonal antibody therapy, and intensive chemotherapy consolidation approaches, in an attempt to improve disease control whilst reducing the requirement for whole-brain radiotherapy. Outcomes for patients that are older, immunocompromised, or have relapsed/refractory disease remain unsatisfactory and there is a paucity of clinical trial data to guide treatment of these groups. This review highlights recent advances in pathobiology, imaging, and clinical management of PCNSL and looks ahead to research priorities for this rare and challenging lymphoid malignancy.

## Introduction

Primary central nervous system lymphoma (PCNSL) is a rare form of non-Hodgkin lymphoma (NHL) comprising 2.2 % of all central nervous system (CNS) tumours [1]. It encompasses lymphoma exclusively involving the brain, spinal cord, eyes, meninges, and cranial nerves, with 90–95 % classified histologically as diffuse large B-cell lymphoma (DLBCL). The majority of PCNSL are sporadic and the incidence increases with age. A minority are attributable to immunosuppressed states, including HIV infection or iatrogenic immunosuppression following organ transplantation. In the era of effective combined antiretroviral therapy (cART), the frequency of HIV-associated PCNSL has diminished [2]. The involvement of critical sites within the CNS presents both diagnostic and therapeutic challenges, with outcomes consistently inferior to systemic DLBCL. Neurocognitive dysfunction and impaired performance status are frequent at clinical presentation, whilst histological confirmation is inherently risky and often yields small tissue biopsies. Moreover, choice of cytotoxic therapy is limited by the inability of many drugs employed for systemic NHL treatment to penetrate the blood–brain barrier (BBB) efficiently. Since the initial description of PCNSL in 1975 [3], treatment algorithms have evolved from whole-brain radiotherapy (WBRT) as a single-modality treatment towards a multi-agent, high-dose methotrexate (MTX)-based, chemotherapy approach where WBRT is reserved for consolidation or for relapsed disease. Given the rarity of PCNSL, together with challenges conducting clinical trials in this patient group, data from randomised studies are scarce and the level of evidence to guide therapeutic decisions is often low. This review covers recent advances in our understanding of biological and clinical aspects of PCNSL, chiefly primary cerebral DLBCL, and potential implications for clinical practice.

## Diagnosis

The diagnosis of CNS lymphoma can be a particular challenge because of lesional response to corticosteroids and MRI features that are shared with other pathologies. The majority of PCNSL are diagnosed via stereotactic biopsy or, less commonly, by flow cytometric analysis of cerebrospinal fluid (CSF) lymphocytes. The conventional approach has been to avoid surgical resection given the risk of neurological sequelae and lack of therapeutic benefit [4]. However, a recent unplanned secondary analysis of the G-PCNSL-SG-1 trial has challenged this view, describing an apparently superior progression-free survival (PFS) for those undergoing complete or subtotal resection [5]. However, this study had a number of limitations, and independent verification in a well-designed and controlled study would be required to change practice.

Rubenstein et al. recently evaluated the utility of CXCL13 (a mediator of B-cell migration) and IL-10 as diagnostic biomarkers with the ability to discriminate CNS lymphoma from other CNS [6•]. The mean concentration of CXCL13 protein in CSF from newly diagnosed PCNSL and SCNSL was >50-fold higher than in CSF from patients without CNS lymphoma (p < 1 × 10^−7^). The concentration of IL-10 in CSF from PCNSL and SCNSL patients was also markedly elevated compared with non-lymphoma comparators (p < 2.3 × 10^−5^). Notably, for patients with PCNSL, both CXCL13 and IL10 levels below the median were associated with significantly longer PFS, although statistical independence from pre-existing clinical risk scores was not shown. The positive predictive value of CXCL13 and IL-10 elevation in CSF was 95 % in the identification of newly-diagnosed HIV-negative PCNSL, with an 88 % negative predictive value [6•]. These interesting findings potentially offer the opportunity for CNS lymphoma diagnosis without brain biopsy, particularly where tissue biopsy is deemed high-risk or of low diagnostic yield. The precision and reproducibility of the diagnostic cut-offs, however, will need to be prospectively evaluated.

Magnetic resonance imaging (MRI) is the principal modality for the detection and monitoring of CNS lesions and recent publications have focussed on the diagnostic and prognostic role of advanced MRI techniques. Cellular density is higher and vascularity is reduced in PCNSL compared to other CNS malignancies, which is reflected in lower apparent diffusion coefficient (ADC) and relative cerebral blood flow (rCBV) values on diffusion-weighted and perfusion MRI, respectively. In support of a prior report suggesting that ADC values are predictive of outcomes in PCNSL [7], a recent study of 23 patients showed that those with baseline ADC_min_ <384 × 10^−6^ mm/s had inferior PFS and overall survival (OS) [8]. A study by the same group reported that low baseline rCBV predicted inferior OS in a small cohort of 25 patients. Patients with both low ADC_min_ and low rCBV had the worst outcomes with 0 % OS at five years compared to 100 % for those with high values for both [9]. A multi-centre phase II study failed to identify ADC as an independent prognostic factor, but numbers were small (n = 28) and the two-year PFS was lower in those with an ADC below the median (57 % vs. 86 % p = 0.27) [10•].

Whole-body ^18^FDG PET-CT has an increased sensitivity for the detection of systemic DLBCL over conventional CT staging [11], and has an important role in the exclusion of systemic lymphoma at presentation. PCNSL lesions characteristically exhibit homogeneous, high-avidity ^18^FDG uptake [12], and one small study has suggested that this may assist in differentiating PCNSL from other intracranial malignancies where MRI findings are equivocal [13]. Pre-imaging corticosteroid therapy is a potential confounding factor, however, and the additional diagnostic value of ^18^FDG PET over modern MRI brain imaging remains poorly defined [12]. The prognostic impact of pre-treatment ^18^FDG PET was evaluated in a retrospective study of 42 patients by Kasenda et al., demonstrating inferior OS on multivariate analysis (p = 0.018) for patients with increased ^18^FDG activity relative to cerebellar uptake at diagnosis [14], consistent with data from an earlier small (n = 17) study [15].

## Molecular

Improved characterisation of PCNSL genotype and phenotype, albeit from small studies with restricted availability of diagnostic material, has the potential to provide prognostic information and identify key molecular pathways that may serve as potential targets for novel therapeutics [16]. An activated B-cell like phenotype is typical (95 % MUM-1+, 50–80 % BCL6+, 10 % CD10+), but evidence of ongoing somatic hypermutation and the preservation of an open reading frame suggests ongoing germinal centre exposure. Therefore, PCNSL does not neatly conform to either of the principal molecular profiles identified in systemic DLBCL, namely germinal centre and activated B-cell subtypes, and appears to exhibit unique transcriptional features by gene expression profiling [17]. In contrast to systemic DLBCL, high expression of BCL-2, BCL-6, and MYC by immunohistochemistry is frequent (70 % of cases studied [18]) and it has been speculated that this may contribute to the adverse prognosis of PCNSL. Recently, the only multi-centre trial to prospectively evaluate PCNSL biomarkers demonstrated that BCL-6 expression, but not MYC, correlated with inferior survival [19••]. Whilst some studies support this finding [20, 21], other retrospective analyses found that BCL-6 overexpression correlated with superior outcomes [22, 23]. Heterogeneous treatment approaches, sample size, and variable methodologies or cut-offs may explain these discrepant findings.

The most frequent genomic aberration identified in PCNSL tissue is deletion of 6p21 involving the HLA locus (56–79 %) [24], a lesion found commonly in DLBCL arising in immune-privileged sites [25], and represents a potential mechanism for immune escape. Deletions within the 6q22-23 region (34–50 %), which contains numerous tumour suppression genes [20, 24, 26] and homozygous silencing/deletion of the cell cycle regulator CDKN2A (45–64 %) [24, 27] have apparent adverse prognostic significance. The MyD-88 L265P activating mutation appears to be a common molecular aberration identified (38–50 %), but no impact on clinical outcome has been demonstrated [21, 24]. The resultant activation of the NFκB pathway, which is also upregulated by less frequent mutations or amplification of MALT1 [28], CARD11 [29], PRDM1 [21, 30], and TBL1XR1 [24], highlights a key survival pathway and potential therapeutic target. Other dysregulated signalling pathways of potential significance include B-cell receptor signalling, with CD79B mutations in 20 % [31, 32], and the JAK/STAT pathway [6•, 33]. Most of the available data, however, has emerged from relatively small studies, thus the frequency and prognostic significance of most individual genomic aberrations requires further validation, preferably in the context of prospective clinical trials.

## Treatment

### Remission Induction

Chemotherapy regimens incorporating HD-MTX are considered the standard of care as induction therapy for newly-diagnosed PCNSL, achieving high rates of initial response when combined with other agents. There is general consensus that MTX should be administered as a rapid infusion (2–4 hours) at a dose of at least 3 mg/m^2^ to maximise therapeutic CSF concentrations, at an interval of 14–21 days [34]. Higher absolute MTX exposure (area under the plasma concentration-time curve, AUC_MTX_, >980–1,100 μmol · h/l) correlated with superior outcomes in two post-hoc and retrospective studies [35, 36], but these findings have not been reproduced in all studies [37]. In patients older than 70 years, a retrospective study found that exceptionally high AUC_MTX_ values (>2,126 μmol · h/l) inversely correlated with OS on multivariate analysis, without evidence of increased treatment toxicity [38]. Given significant inter-personal variations in MTX metabolism, an individualised dosing schedule based on pharmacokinetic analysis rather than body surface area has been suggested [39], but not prospectively validated. Recent reports that polymorphisms in key genes involved in MTX metabolism can influence outcomes and toxicity in adult lymphoma [40] and paediatric acute lymphoblastic leukaemia [41] warrant further investigation in PCNSL.

Modern protocols typically employ between four and eight cycles of HD-MTX-based therapy but comparative data on treatment duration is lacking. Historical studies of HD-MTX response have suggested minimal additional radiological response to HD-MTX beyond two cycles [42], but this phenomenon is likely to be influenced by a number of factors, including partner chemotherapy agents. In the randomised, phase II, multi-centre IELSG-20 trial, 75 % of maximum responses were achieved following the first two cycles of treatment. However, for patients achieving a PR, further tumour response was observed in patients receiving combination chemotherapy (HD-MTX/Ara-C, 10/18 patients) but not HD-MTX monotherapy [43]. In a recent trial of rituximab, HD-MTX, procarbazine and vincristine (R-MPV), CR rates improved from 47 % after five cycles of chemotherapy to 79 % after seven [10•]. More information on the kinetics of PCNSL response in the setting of modern immunochemotherapy protocols is required to inform the optimal duration of induction regimens.

### Combination Chemotherapy

To improve on outcomes with single-agent HD-MTX, the IELSG20 trial assessed the role of combined antimetabolite therapy, with HD-MTX and cytarabine [43]. This study demonstrated a superior CR rate of 46 % compared to HD-MTX alone (18 %, p = 0.006), with significant improvements in PFS but not OS (three-year OS 46 % vs. 32 %, p = 0.07). Although a relatively small study (n = 79), it remains the first randomised trial of combination chemotherapy in PCNSL to complete accrual. Several studies have evaluated the additional value of BBB-penetrating alkylating agents (such as temozolomide, procarbazine, and thiotepa), providing non-cross resistant agents that, unlike antimetabolite chemotherapy, are also cytotoxic to cells in G0 of the cell cycle. Rubenstein et al. used methotrexate, temozolomide, and rituximab (MT-R) induction followed by etoposide and cytarabine (EA) consolidation in a prospective multi-centre trial of 44 patients, achieving a two-year PFS of 57 % and four-year OS of 65 % [19••]. A recent multi-centre phase II trial reported promising outcomes with 52 patients treated with R-MPV induction therapy followed by reduced or standard dose WBRT (23.4Gy (n = 31) or 45Gy) and cytarabine consolidation [10•], achieving median PFS and OS of 3.3 and 6.6 years, respectively. The addition of thiotepa to HD-MTX and cytarabine was piloted in a small multi-centre study (n = 20), with inferior results compared to the IELSG20 trial, attributed to a 50 % protocol reduction in cytarabine dose (1 g/m^2^ for four doses) [44], although the optimal thiotepa dose in this setting has not been ascertained. The role of thiotepa is currently being evaluated in the ongoing, randomised IELSG32 study (EudraCT number 2009-012432-32). Although the outcomes of the studies discussed above appear at least comparable to those from IELSG20, meaningful comparison across studies is inherently limited due to differences in study design, patient characteristics, and the range of consolidation approaches. Whilst it is clear that multi-agent chemotherapy is superior to MTX alone, in the absence of further RCT evidence the optimal combination remains uncertain.

### Rituximab

In contrast to the survival advantage witnessed in systemic DLBCL, the benefit of combining rituximab with chemotherapy for PCNSL remains unclear. Rituximab has limited CNS penetration with intravenous administration, achieving 0.1–4.4 % of plasma concentrations [45]. Single-agent efficacy was demonstrated in 12 patients with refractory/relapsed PCNSL with radiographic response to intravenous rituximab monotherapy in 36 % [46] and encouraging response rates have been achieved with rituximab 375–500 mg/m^2^ in conjunction with combination chemotherapy in single-arm trials [8, 10•, 19••, 45, 47]. Two recent studies have retrospectively compared immunochemotherapy outcomes against historical chemotherapy regimens to extrapolate the relative benefit of rituximab. Although the quality of responses appears to be improved with the addition of rituximab, no survival advantage has yet been demonstrated on multivariate analysis [48, 49]. Results from ongoing randomised studies (IELSG32: NCT01011920, and HOVON 105 PCNSL/ALLG NHL24 trial: EudraCT 2009-014722-42) are essential to define the role of rituximab in induction therapy for PCNSL.

### Intrathecal Therapy

Detectable meningeal disease is present in 15 % at diagnosis [50] and may serve as a reservoir for PCNSL cells. The presence of meningeal disease does not appear to have a prognostic impact, irrespective of whether intrathecal therapy is employed [50–52]. The additional benefit of intrathecal therapy remains unclear given the ability of HD-MTX to achieve therapeutic concentrations within the CSF [53]. Two recent non-randomised studies have suggested higher rates of early relapse with omission of intrathecal therapy in comparison with otherwise identical polychemotherapy regimens [54, 55]. Several other studies, however, have failed to demonstrate a clear improvement in response rates or OS with intrathecal therapy, leading to omission of intrathecal treatment from many current chemotherapy regimens [51, 56]. In light of the limited ability of immunotherapy to cross the BBB, however, the role of CSF-directed therapy needs to be re-evaluated. Notably, a phase I study of intraventricular rituximab/methotrexate in 14 patients with isolated CNS lymphoma relapse, including six PCNSL patients, reported ORR and CR rates of 75 % and 43 %, respectively [57•].

### Consolidation Treatment

#### WBRT

Following introduction of HD-MTX-based chemotherapy, WBRT (36-45Gy) has continued to be employed to consolidate responses and provide more durable disease control. Delayed neurotoxicity [58, 59•], however, is a major limitation that is clinically evident in approximately one-third of patients, particularly with increasing age, and associated with significant morbidity and mortality. The only phase III trial thus far to complete accrual in PCNSL aimed to demonstrate that omission of consolidation WBRT after MTX-based chemotherapy resulted in non-inferior OS rates. Although outcomes suggested that WBRT can be safely omitted in selected patients achieving CR with induction chemotherapy, this ambitious study failed to meet its primary endpoint and major limitations in study design, amendments, and protocol adherence limit interpretation of these data [58]. A recent systematic review, which assessed outcomes of chemotherapy versus combined modality treatment using a decision-analytic model, has suggested improvements in both survival and quality-adjusted life years with consolidation WBRT for those <60 years only [60]. The emerging outcome data for HDT-ASCT consolidation for younger, sufficiently fit patients (described below), however, challenges this approach. Deferring WBRT in patients achieving CR with chemotherapy is a particularly attractive concept for those >60 years.

In an attempt to ameliorate the long-term neurocognitive sequelae of WBRT at standard doses, investigators have assessed the value of reduced dose WBRT (rdWBRT). Inferior outcomes have been described with a reduced consolidation WBRT dose (30.6Gy) following CHOD/BVAM induction therapy in a non-randomised comparison [61]. Morris et al. recently reported encouraging rates of disease control using 23.4Gy radiotherapy as consolidation therapy following the R-MPV protocol, with a PFS of 7.7 years for the selected subgroup (n = 31) achieving CR with immunochemotherapy. Prospective neuropsychological evaluation demonstrated no overall cognitive decline, in 12 patients assessed 48 months after rdWBRT [10•]. The PFS for the whole cohort (n = 52), however, was 3.3 years in this single-arm study. Importantly, a randomised study of R-MPV versus R-MPV-rdWBRT is planned (RTOG 1114).

#### Chemotherapy Consolidation

The efficacy of high-dose chemotherapy and autologous stem cell transplantation (HDT-ASCT) was first demonstrated in the context of relapsed/refractory PCNSL [62] and has been subsequently studied as a consolidation approach in first-line therapy [63] in younger, fit patients. The concept of eradicating minimal residual disease by the use of myeloablative doses of non-cross resistant agents that efficiently penetrate the BBB is supported by non-comparative phase II clinical trial outcomes. Thiotepa is a small, lipophilic polyfunctional alkylator that efficiently penetrates the BBB and has been incorporated as a key agent in HDT-ASCT protocols for PCNSL. Schorb et al. retrospectively analysed outcomes from the largest reported cohort (n = 105) treated with thiotepa/BCNU HDT-ASCT first-line consolidation following HD-MTX-based induction. The median age was 54 and median KPS was 80 %. HDT-ASCT-related mortality was 2.8 %, and 36 % of patients received additional WBRT. Outcomes were encouraging, with a five-year OS of 79 % and a median PFS and OS of 85 and 121 months, respectively [64•]. On an intention-to-treat basis, long-term follow-up data was recently reported for some of these patients included in two prospective single-arm phase II trials involving HD-MTX and HD-Ara-C/thiotepa induction followed by carmustine/thiotepa-conditioned ASCT, with (n = 30) or without (n = 13) WBRT. A total of 34 of 43 patients proceeded to ASCT and median OS for the whole cohort was 104 months with a five-year OS of 70 % [65]. In most studies, evidence of chemosensitivity to agents employed in induction or salvage regimens is not an absolute prerequisite to proceed to HDT-ASCT. Long-term remissions have been demonstrated in patients who failed to achieve PR with HD-MTX-based induction prior to HDT-ASCT, although outcomes in this group as a whole are inferior [64•, 66]. No direct comparison of conditioning regimens in PCNSL has been conducted, however, earlier results using BEAM (carmustine, etoposide, cytarabine, melphalan) have been disappointing, with a PFS of 9.3 months in one study (n = 14) [67].

Other groups have investigated dose-intensive, non-myeloablative, consolidation chemotherapy as an alternative strategy. A multi-centre prospective trial of MT-R induction followed by EA consolidation resulted in a median PFS of 28 months and four-year OS of 65 % [19••]. Notwithstanding a relatively high rate of early disease progression, disease control using this chemotherapy consolidation strategy appears at least comparable with chemoradiation protocols. These results need further validation in an independent cohort. This strategy is potentially attractive in older or unfit patients, who are ineligible for HDT-ASCT and have higher rates of neurotoxicity with WBRT. Data from a phase II study, presented in abstract form, demonstrated the feasibility of HD-Ara-C (3 g/m^2^/d for two days) as a consolidation approach for patients with a median age of 72 years [68]. Response-adapted consolidation based on the results of interim response assessment is another potential approach; a small retrospective study (n = 40) did not identify a difference in outcome with omission of WBRT or ASCT in those achieving early CR at interim assessment (n = 10) [69], but requires prospective validation.

Efficacy and toxicity data from a number of ongoing randomised trials in Europe and the US comparing thiotepa-based HDT-ASCT with WBRT (IELSG32: NCT01011920), or non-myeloablative chemotherapy (CALGB 51101: NCT01511562), should help inform the optimal consolidation strategy in PCNSL.

### Salvage

Currently, there exists no standard treatment approach for patients with relapsed and refractory PCNSL. Published data are largely restricted to small, uncontrolled, retrospective studies, in which outcomes are influenced by a number of parameters including prior therapies and response durations. With these caveats in mind, the overall response rates reported from both prospective and retrospective studies are typically 10–40 %. A recent retrospective analysis of MTX re-challenge described high response rates amongst 39 patients re-treated at a median of 26 months from initial diagnosis with MTX-based therapy [70]. For patients with early relapse or refractory disease, a salvage regimen employing non-cross-resistant chemotherapy regimens is rational. Mappa et al. recently reported a retrospective study (n = 22) of salvage therapy with rituximab, ifosfamide, and etoposide for refractory (n = 11) or relapsed PCNSL; in this high-risk patient group the ORR was 41 % with a two-year OS of 25 % [71]. In studies where eligible patients have proceeded to HDT-ASCT, available data support the use of thiotepa-based HDT-ASCT in relapsed PCNSL, resulting in five-year EFS and OS rates of 37.8 % and 51.4 %, respectively, in the largest study to date (n = 79) [66]. Results of WBRT as a salvage treatment are equivalent to those reported with many salvage chemotherapy regimens, with a PFS of 10–10.8 months, but are rarely durable [72, 73]. WBRT is most often employed as consolidation following salvage chemotherapy or as a palliative single-modality treatment.

Temozolomide has been evaluated in a number of retrospective [74–76] and prospective [77, 78] studies, with and without rituximab, demonstrating modest efficacy and some durable responses (ORR 14–53 %, one-year OS 31–71 %) but disappointing PFS of <2-2.8 months. Use of pemetrexed as an alternative antifolate agent has been reported in two small cohorts of heavily pre-treated patients with a median PFS of 5.7–5.8 months and one-year OS of 45 % [79, 80] and studies addressing its efficacy are ongoing. Other agents showing limited single-agent efficacy in heavily pre-treated patients include bendamustine [81] and topotecan [82]. There is interest in assessing the efficacy of other drugs, such as lenalidomide (NCT01956695), pomalidomide (NCT01722305), temsirolimus (NCT00942747), ibrutinib, and other agents targeting B-cell signalling [16]. Given that a significant proportion of patients will not be fit for or not respond to salvage chemotherapy, there is an urgent need for the assessment of novel agents or treatment combinations in the context of clinical trials.

### Older Patients

With the median age at diagnosis of PCNSL rising above 63 years [83, 84] and over 20 % patients older than 80 years [85], establishing optimal treatment in older patients is of paramount importance. The true incidence may be underestimated if there is reluctance to biopsy cerebral lesions in this age group, as the possible treatment options are considered to be limited. Symptoms can be misinterpreted in older patients resulting in significant treatment delays [86]. Altered metabolism of cytotoxic drugs [38] and impaired organ function [83] present additional therapeutic challenges.

Age is consistently reported as a principle adverse prognostic factor in PCNSL. Defining the ‘older patient’ is problematic, however, as physiological fitness does not always equate to chronological age and published studies have applied different age thresholds. In practical terms, this cohort is often defined by the ability to tolerate therapies such as WBRT (<60 years) or HDT-ASCT (<70-75 years). The G-PCNSL-SG-1 trial reported a median OS of 12.5 months in those older than 70 years (versus 26.6 months for younger patients), with a marked difference in PFS for those in CR after induction therapy (16.1 vs. 35 months p = 0.024) not explained by WBRT-related neurotoxicity alone [87]. The use of WBRT results in high rates of clinically significant neurotoxicity in patients over 60 years [88]. Consequently, WBRT is often avoided in those achieving CR with induction chemotherapy. WBRT offers only modest efficacy as sole therapy, with a median OS of 5–12 months [89], but may offer a palliative approach for some patients. Population-based studies, however, suggest a reluctance to use HD-MTX protocols in older patients, with 36–39 % receiving WBRT alone [85, 89]. In a cohort of 22 patients aged 80–90 years, HD-MTX was well-tolerated, with only two patients requiring dose reductions and a two-year OS of 33 % [86]. By contrast, a population-based study (n = 40) reported that 25 % of patients >60 years were unable to tolerate more than one cycle of MTX-based chemotherapy due to renal, haematological, or pulmonary toxicity, with an additional two treatment-related deaths (5 %) [89].

A number of studies have focused on the efficacy of chemotherapy without WBRT in older patients. A randomised phase II study, published in abstract form, randomised 98 patients (median age 72 years, median KPS 70 %) to primary treatment with either MT (MTX-temozolomide) or MPV-A (methotrexate, procarbazine, vincristine, cytarabine). Efficacy endpoints favoured MPV-A (median OS 31 versus 14 months p = 0.2) with no increase in toxicity [68], although the study lacked the power to demonstrate statistical superiority. A phase II trial of chemoimmunotherapy with MTX, lomustine, procarbazine, and rituximab in 28 patients reported a median OS of 17.5 months [47], similar to the median OS of 15 months reported in a preceding trial without rituximab [90]. Omission of lomustine from the regimen has resulted in improved tolerability (G. Illerhaus, Stuttgart, personal communication, 2014) and formal reporting of the outcome is awaited.

### PCNSL in People Living With HIV

Immunodeficiency-associated PCNSL is a distinct clinical entity characterised by Epstein Barr virus (EBV)-positivity and the presence of multifocal, often necrotic lesions on gadolinium-enhanced MRI. Incidence inversely correlates with CD4 counts and, historically, survival was strongly influenced by HIV-related comorbidities. In the cART era, the frequency of HIV-associated PCNSL has fallen whilst survival has improved [91], although outcomes remain inferior to those of immunocompetent patients [92]. Surrogate measures are often used for diagnosis, using a combination of thallium-201 SPECT scanning and presence of EBV in the CSF [93, 94], therefore availability of diagnostic material for biologic studies is limited. With increasing use of intensive treatment regimens, however, histological diagnosis will have a greater influence on choice of treatment and should be obtained where feasible.

Given that HIV seropositivity is typically an exclusion criterion for prospective PCNSL studies, therapy has been empirically adopted from treatment of PCNSL in immunocompetent patients, akin to the paradigm of systemic lymphoma therapy [95]. Case reports of isolated responses to cART alone are few [96] and subject to selection bias, therefore, it is not recommended as the sole treatment modality. Use of either WBRT or chemotherapy independently reduced the risk of death in a large retrospective HIV-PCNSL cohort [91]. HD-MTX-based regimens have been tolerated in immunodeficient patients, but trials in the cART era are lacking [97] and data is particularly scarce on patients receiving combination chemotherapy [92, 98]. There is an isolated case report of successful ASCT [99]. Despite this, in large population-based studies, reasonable survival rates of 54 % at one year [100] and 22.8 % at five years [101] were reported.

### Post-Treatment Evaluation and Monitoring

Treatment-related neurotoxicity occurring months to years following completion of therapy is frequently a progressive condition that can be disabling, even fatal, in some patients. The risk of neurotoxicity correlates with the dose of WBRT and increasing age [102, 103]. Whether combination chemotherapy confers additive risk of neurotoxicity is not yet clear, largely due to the paucity of prospective psychometric assessment in many trials. A standardised battery of neuropsychological and quality of life assessments have been published and are being incorporated into ongoing prospective studies [103]. Recently, Doolittle et al. [59•] reported on the long-term cognitive outcomes in 80 PCNSL survivors who achieved CR with HD-MTX-based protocols, of which 19 % incorporated WBRT at a dose of 45Gy or greater. At a median follow-up of 5.5 years, 47 % of patients treated with chemoradiation protocols demonstrated impairment in multiple cognitive domains, compared to 9–16 % receiving chemotherapy alone (p = 0.0237). Correa et al. presented cognitive assessment on 50 patients, 24 of whom received WBRT plus chemotherapy and 26 who received chemotherapy alone. Quality of life was lower in WBRT patients, with significant reductions throughout all cognitive domains compared to age-matched controls [104]. Interpretation of both cross-sectional studies, however, is limited by lack of formal assessment of pre- and post-treatment cognitive status. Almost all patients with delayed neurotoxicity have marked white matter changes on MRI [102], and the extent of white matter changes has been shown to be correlated inversely with cognitive function in some studies [59•] but not others [104]. This is a common post-treatment finding in PCNSL, however, and is not an adequate surrogate for formal psychometric assessment.

A large, single-centre retrospective (n = 209) of PCNSL patients monitored with post-treatment brain imaging every 4–6 months demonstrated that, of 124 patients in CR, 80 % of relapses that occurred were symptomatic and detected between surveillance scans [105•]. Given the incidence of late relapses and lack of evidence for early salvage treatment, there is no clear indication for surveillance imaging outside of clinical trials.

Relatively few studies have reported survival rates beyond five years, with varying incidence of late relapse and limited evidence for emergence of a survival plateau. Relapses can occur more than 10 years after initial diagnosis, with evidence of clonal persistence [106]. In a single-centre retrospective cohort, of those achieving CR with induction therapy (n = 268), 10 out of 48 patients in ongoing remission at five years subsequently relapsed (20.8 %) [106]. Prospective follow-up of 41 patients treated with chemoradiotherapy at a median of 12 years has suggested the emergence of a survival plateau with a 10-year OS of 24 % [107]. Long-term follow-up of patients in two trials involving ASCT consolidation (n = 43) reported that 50 % of relapses (6/12) occurred more than five years post-treatment [65] and emphasises the need for long-term follow-up in PCNSL survivors to fully evaluate treatment efficacy.

## Conclusions

Information from randomised clinical trials is essential to further improve the management of PCNSL and answer critical questions such as the optimal induction chemotherapy and consolidation strategy. Correlative biological studies in the context of prospective trials will refine our knowledge of PCNSL biology and potentially enable identification of those with poor-risk disease at diagnosis, for whom intensification of treatment and/or novel therapeutics may be of benefit. Focussing on neurocognitive outcomes and long-term follow-up of survivors will allow refinement of the balance between treatment toxicity and cure.
